# Iron overload in endometriosis peritoneal fluid induces early embryo ferroptosis mediated by HMOX1

**DOI:** 10.1038/s41420-021-00751-2

**Published:** 2021-11-15

**Authors:** Shishi Li, Yier Zhou, Qiongxiao Huang, Xiaohua Fu, Ling Zhang, Fang Gao, Zhen Jin, Limei Wu, Chongyi Shu, Xirong Zhang, Weihai Xu, Jing Shu

**Affiliations:** Reproductive Medicine Center, Department of Reproductive Endocrinology, Zhejiang Provincial People’s Hospital, Affiliated People’s Hospital, Hangzhou Medical College, 310000 Hangzhou, P. R. China

**Keywords:** Pathogenesis, Infertility

## Abstract

Endometriosis is one of the most common disorders that causes infertility in women. Iron is overloaded in endometriosis peritoneal fluid (PF), with harmful effects on early embryo development. However, the mechanism by which endometriosis peritoneal fluid affects embryonic development remains unclear. Hence, this study investigated the effect of iron overload on mouse embryos and elucidated the molecular mechanism. Iron overload in endometriosis PF disrupted blastocyst formation, decreased GPX4 expression and induced lipid peroxidation, suggesting that iron overload causes embryotoxicity and induces ferroptosis. Moreover, mitochondrial damage occurs in iron overload-treated embryos, presenting as decreased ATP levels, increased ROS levels and MMP hyperpolarization. The cytotoxicity of iron overload is attenuated by the ferroptosis inhibitor Fer-1. Furthermore, Smart-seq analysis revealed that HMOX1 is upregulated in embryo ferroptosis and that HMOX1 suppresses ferroptosis by maintaining mitochondrial function. This study provides new insight into the mechanism of endometriosis infertility and a potential target for future endometriosis infertility treatment efforts.

## Introduction

Endometriosis is the presence of endometrial tissue, including both the glandular epithelium and stroma, outside the uterine cavity [1]. It is one of the most common benign gynecological disorders, affecting 10–15% of all women of reproductive age and a much higher proportion in infertile women, approximately 25–40% [2]. Endometriosis patients may have an abnormal pelvic environment that affects pregnancy. Patients with endometriosis present significantly lower mature oocyte and fertilization rates [3]. Peritoneal fluid (PF) from endometriosis has harmful effects on early embryo development [4–6]. In vivo experiments have also suggested that endometriotic fluid is detrimental to reproductive performance and subsequent blastocyst development [7, 8]. However, the mechanism by which PF affects embryonic development in endometriosis remains unclear.

Several studies have demonstrated the presence of iron overload in the PF of endometriosis patients [9–11]. Iron is essential for the biological processes of organisms, with roles in oxygen transport, energy metabolism, ATP generation, and DNA synthesis and repair [12]. However, iron overload exerts significant cytotoxic effects on living cells via the iron-catalyzed Haber-Weiss reaction [13].

Ferroptosis is a novel form of regulated cell death characterized by the iron-dependent accumulation of lipid peroxides to lethal levels [14, 15]. Iron metabolism plays a critical role in the process [14]. Ferroptosis is also associated with dramatic morphological changes in mitochondria, including mitochondrial fragmentation and cristae enlargement [14, 16], and some potent ferroptosis inhibitors appear to explicitly target mitochondria, confirming the potential involvement of mitochondria in ferroptosis [17]. Additionally, mitochondrial damage plays a key role in the pathogenesis of infertility caused by endometriosis [18, 19].

In the present study, we found that iron overload in the PF of endometriosis causes embryotoxicity and triggers ferroptosis. By Smart-seq, we identified that HMOX1 is upregulated in embryo ferroptosis and that HMOX1 suppression protects against ferroptosis by maintaining mitochondrial function.

## Results

### Iron overload in the PF of endometriosis patients

**Fig. 1:**
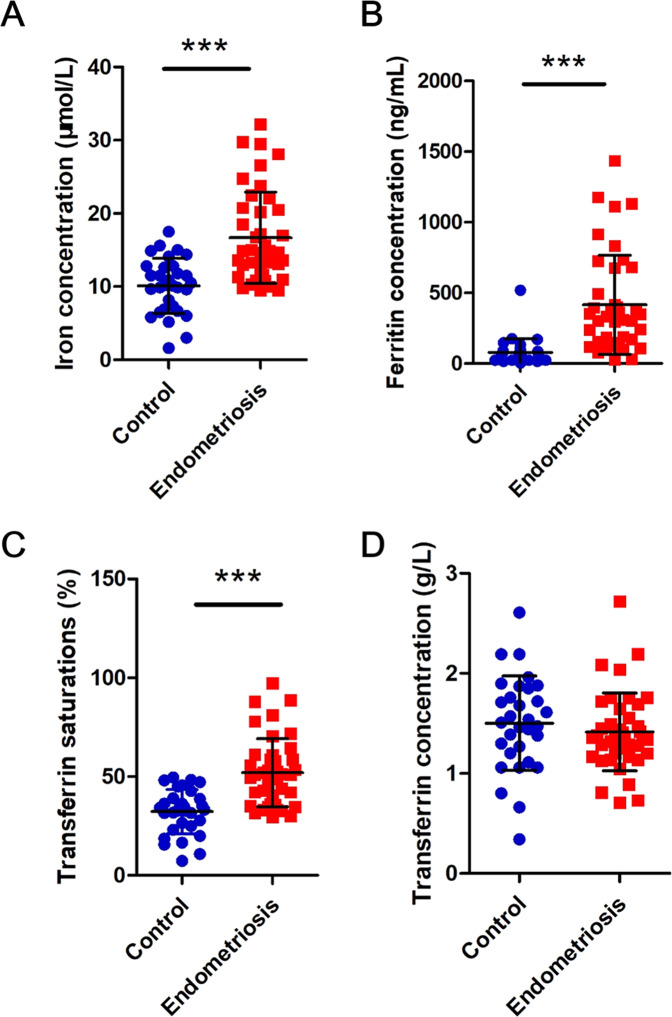
Iron overload in peritoneal fluid of endometriosis patients. **A** Iron concentration, **B** ferritin concentration, **C** transferrin saturation, **D** Transferrin concentration. Control (*n* = 31) and endometriosis (*n* = 41). ***P < 0.001.

To determine the level of iron in endometriosis PF, we measured the iron, transferrin, and ferritin concentrations in the PF supernatant and calculated the transferrin saturations. Clinical information on the endometriosis and control patients was summarized in Table 1. The PF of patients with endometriosis (*n* = 41) showed significantly higher iron concentrations (16.67 vs. 10.13 μmol/L; *P* < 0.001), ferritin concentrations (416.6 vs. 79.10 ng/mL; *P* < 0.001) and transferrin saturation (51.93% vs. 32.25%; *P* < 0.001) than those of the controls (*n* = 31; Fig. 1A–C). The peritoneal transferrin concentrations did not differ between women with and without endometriosis (1.41 vs. 1.52 g/L; *P* = 0.39; Fig. 1D).

**Table 1:** Clinical description of endometriosis and control patients.

Age	Control (*n* = 31)	Endometriosis (*n* = 41)	*P* value
	33.74 ± 9.04	32.44 ± 5.78	<0.05
*Menstrual cycle phase*			
Proliferative	21	27	>0.05
Secretory	10	14	>0.05
Surgical indications	Tubal infertility (12/31)	Ovarian endometriosis (4/41)	
	Mesosalpinx cyst (14/31)	Peritoneal endometriosis (18/41)	
	Uterine Leiomyoma(5/31)	Peritoneal and Ovarian endometriosis (18/41)	
		Peritoneal and deep endometriosis (1/41)	
Revised ASRM	–	Stage I–II (1–15):(19/41)	
		Stage III (16–40): (13/41)	
		Stage IV (40): (9/41)	

*ASRM* American Society for Reproductive Medicine

### Endometriosis PF induces ferroptosis in mouse embryos

**Fig. 2:**
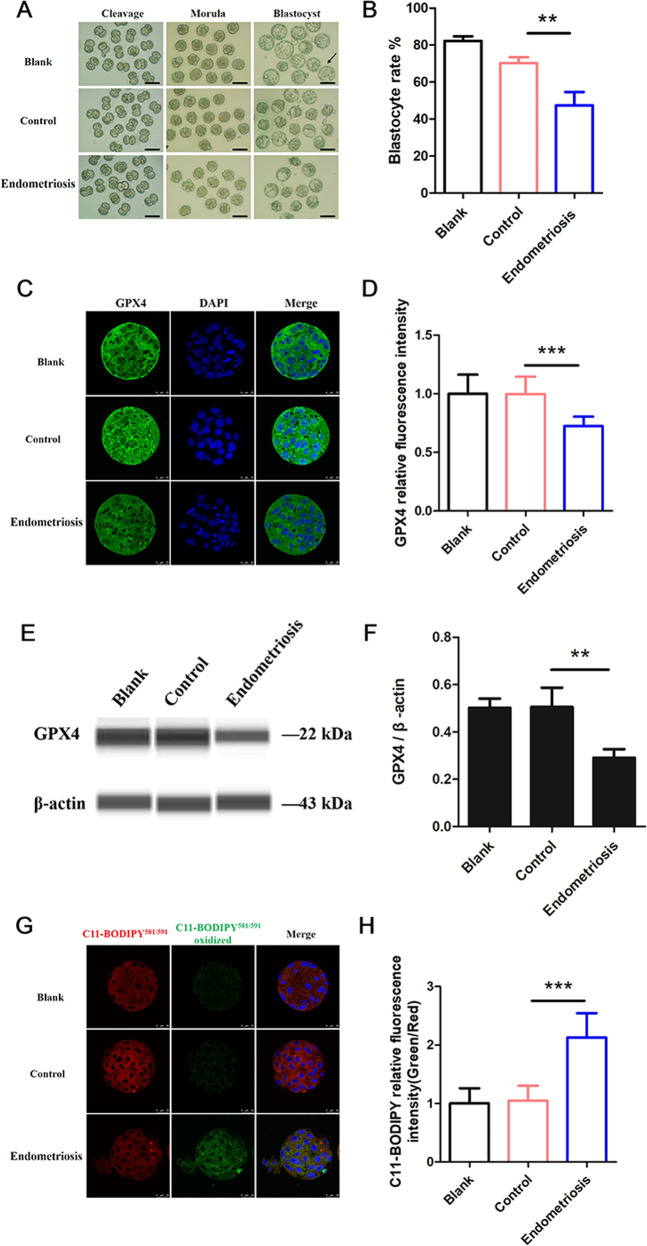
Endometriosis PF induces ferroptosis in mouse embryos. **A** The morphology of mouse embryos cultured with control PF and endometriosis PF at various developmental stages, blank refers to mouse embryos cultured with only medium, the scale bar = 100 μm, the black arrow indicates the blastocyst. **B** The blastocyst rate of mouse embryos cultured with endometriosis PF and control PF. **P < 0.01. **C** Representative immunostaining for GPX4 in mouse embryos cultured with endometriosis PF and control PF. Nuclei were stained blue with DAPI, the scale bar = 25 μm. **D** Relative fluorescence intensity statistics of GPX4 in blank (*n* = 16), control (*n* = 11) and endometriosis (*n* = 13). Data are expressed as mean ± SD. ***P < 0.001. **E** Simple western immunoblots showed GPX4 expression in mouse embryos from blank, control, and endometriosis. β-actin is shown as loading control. **F** Quantification analysis of GPX4 expression in embryos from each treatment, (*n* = 3). Data are expressed as mean ± SD. **P < 0.01. **G** The lipid peroxidation stained by BODIPY 581/591 C11 in mouse embryos cultured with endometriosis PF and control PF. Red representing non-oxidized BODIPY 581/591 C11, and green representing oxidized BODIPY 581/591 C11, the scale bar = 25 μm. **H** Relative fluorescence intensity statistics of BODIPY 581/591 oxidation (Green/Red) in blank (*n* = 20), control (*n* = 20) and endometriosis (*n* = 22). Data are expressed as mean ± SD. ***P < 0.001.

To clarify the effect of endometriosis PF on embryos, we cultured mouse cleavage embryos with PF from patients with and without endometriosis and observed the morphology at the cleavage, morula and blastocyst stages (Fig. 2A). The endometriosis group had a significantly lower blastocyst rate than the control group (Fig. 2B). Mouse embryos cultured with endometriosis PF exhibited decreased GPX4 expression (Fig. 2C, D), which uses glutathione to selectively detoxify lipid hydroperoxides and acts as a gatekeeper for ferroptosis [20]. To further confirm that endometriosis PF can decrease GPX4 expression in embryos, we also conducted automated capillary-based simple western immunoblots to detect the embryonic expression of GPX4. As shown in Fig. 2E, F, exposure of embryos to endometriosis PF resulted in a significant reduction in GPX4 expression, which was consistent with the IF staining results. Additionally, endometriosis PF treatment induced the accumulation of lipid radicals in mouse embryos (Fig. 2G, H). Oxidized BODIPY 581/591 C11 (green) was higher in the endometriosis group than in the control group, confirming the involvement of ferroptotic cell death. Our results imply that endometriosis PF may cause ferroptosis in embryos.

### Iron overload affects embryonic development and induces ferroptosis

**Fig. 3:**
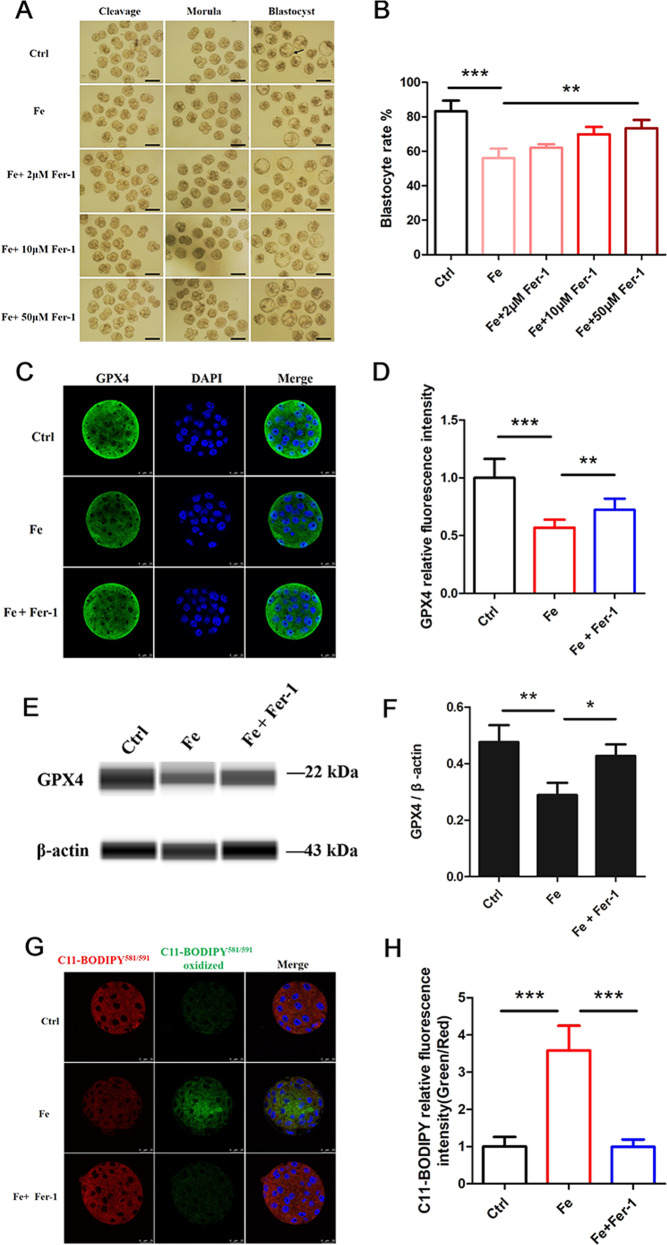
Iron overload affects embryonic development and induces ferroptosis. **A** The morphology of mouse embryos cultured with 100 μM Fe and 100 μM Fe + Fer-1 (2, 10, and 50 μΜ) at various developmental stages, the scale bar = 100 μm, the black arrow indicates the blastocyst. **B** The blastocyst rate of mouse embryos cultured with 100 μM Fe and 100 μM Fe + Fer-1 (2, 10, and 50 μΜ). ***P < 0.001, **P < 0.01. **C** Representative immunostaining for GPX4 in mouse embryos treated with Fe and Fe + Fer-1. Nuclei were stained blue with DAPI, the scale bar = 25 μm. **D** Relative fluorescence intensity statistics of GPX4 in control (*n* = 13), Fe (*n* = 13) and Fe + Fer-1 (*n* = 11). Data are expressed as mean ± SD. **P < 0.01, ***P < 0.001. **E** Simple western immunoblots showed GPX4 expression in mouse embryos from control, Fe and Fe + Fer-1. β-actin is shown as loading control. **F** Quantification analysis of GPX4 expression in embryos from each treatment, (*n* = 3). Data are expressed as mean ± SD. **P < 0.01, *P < 0.05. **G** The lipid peroxidation stained by BODIPY 581/591 C11 in mouse embryos cultured with Fe and Fe + Fer-1. Red representing non-oxidized BODIPY 581/591 C11, and green representing oxidized BODIPY 581/591 C11, the scale bar = 25 μm. **H** Relative fluorescence intensity statistics of BODIPY 581/591 oxidation (Green/Red) in control (*n* = 20), Fe (*n* = 22) and Fe + Fer-1 (*n* = 19). Data are expressed as mean ± SD. ***P < 0.001.

To verify that iron overload in endometriosis PF was the most reason cause embryo ferroptosis. We treated mouse embryos with Fe and the ferroptosis inhibitor Fer-1 and observed the embryo morphology at different developmental stages (Fig. 3A). The results showed that Fe group had a significantly lower blastocyst rate than the control group, but the inhibitory effect was rescued by Fer-1, and the blastocyst rate increased as the concentration of Fer-1 increased (Fig. 3B). Additionally, Fe treatment significantly reduced GPX4 expression, and these effects were rescued by treating the embryos with Fer-1 (Fig. 3C–F). Fe treatment also caused lipid peroxidation accumulation in mouse embryos and could be blocked by Fer-1 (Fig. 3G, H). Our results imply that iron overload impairs embryonic development and induces ferroptosis.

### Iron overload induces mitochondrial impairment

**Fig. 4:**
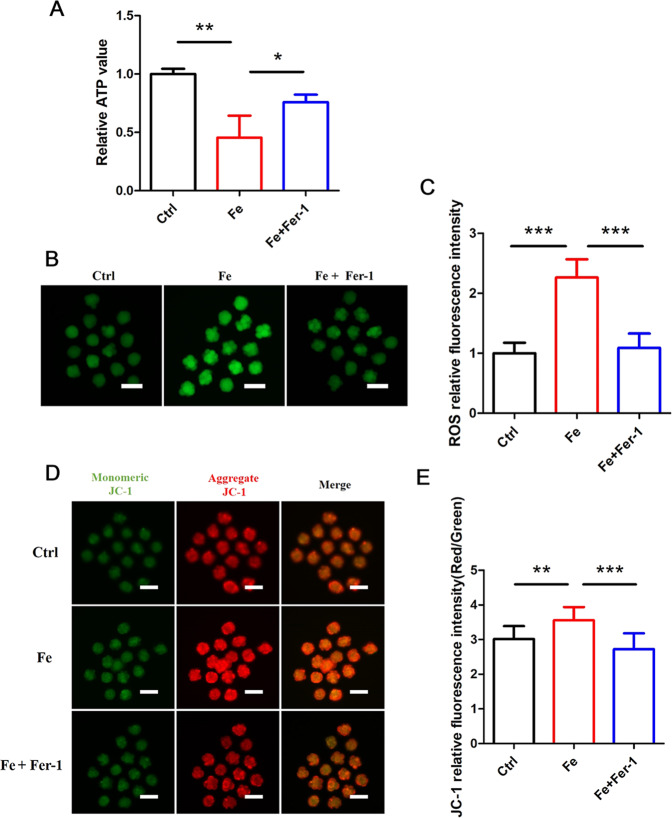
Iron overload caused mitochondrial damage in mouse embryo. **A** ATP of mouse embryos cultured with Fe and Fe + Fer-1 were measured using a bioluminescent assay system. Data are represented as mean ± SD. *P < 0.05, **P < 0.01. **B** Reactive oxygen species (ROS) detection of mouse embryos cultured with Fe and Fe + Fer-1, the scale bar = 100 μm. **C** Relative fluorescence intensity statistics of ROS in control (*n* = 15), Fe (*n* = 14) and Fe + Fer-1 (*n* = 15). Data are expressed as mean ± SD. ***P < 0.001. **D** MMP detection of mouse embryos cultured with Fe and Fe + Fer-1 by using JC-1 staining. When excited at 488/561 nm, JC-1 monomers emit a green fluorescence with a maximum at ~510 nm, and high MMP aggregate JC-1 emits red fluorescence with a maximum at ~590 nm. The scale bar = 100 μm. **E** Relative fluorescence intensity statistics of JC-1 (Red/Green) in the control (*n* = 15), Fe (*n* = 15) and Fe + Fer-1 (*n* = 15). Data are expressed as mean ± SD. **P < 0.01, ***P < 0.001.

We next assessed the direct influence of iron on mitochondrial function. Mitochondria serve as the powerhouse of embryos, where their primary function is to synthesize ATP via oxidative phosphorylation. However, generating ATP through the respiratory chain results in the production of reactive oxygen species as metabolic byproducts. As shown, Fe caused a strong deficiency in ATP production, while Fer-1 reduced this Fe-induced effect by maintaining ATP levels (Fig. 4A). We investigated the total ROS levels using the fluorescent probe DCHFDA (Fig. 4B, C). Treatment of cultured embryos with Fe dramatically increased the total ROS level, while adding Fer-1 reduced the total ROS levels. Furthermore, we evaluated the mitochondrial membrane potential (MMP), which is commonly used to measure mitochondrial function. With high MMP, JC-1 remains in the aggregate form and emits red fluorescence. Based on this analysis, we found that mouse embryos treated with Fe had significantly more aggregate JC-1 (red) than control embryos, which was consistent with MMP hyperpolarization (Fig. 4D, E). Additionally, Fer-1 counteracted this Fe-induced mitochondrial injury, presumably by maintaining mitochondrial ATP and decreasing ROS. These results suggest that iron overload-induced ferroptosis accompanied by mitochondrial damage and ferroptosis inhibition protects mitochondrial function.

### Upregulation of HMOX1 in iron overload induces ferroptosis

**Fig. 5:**
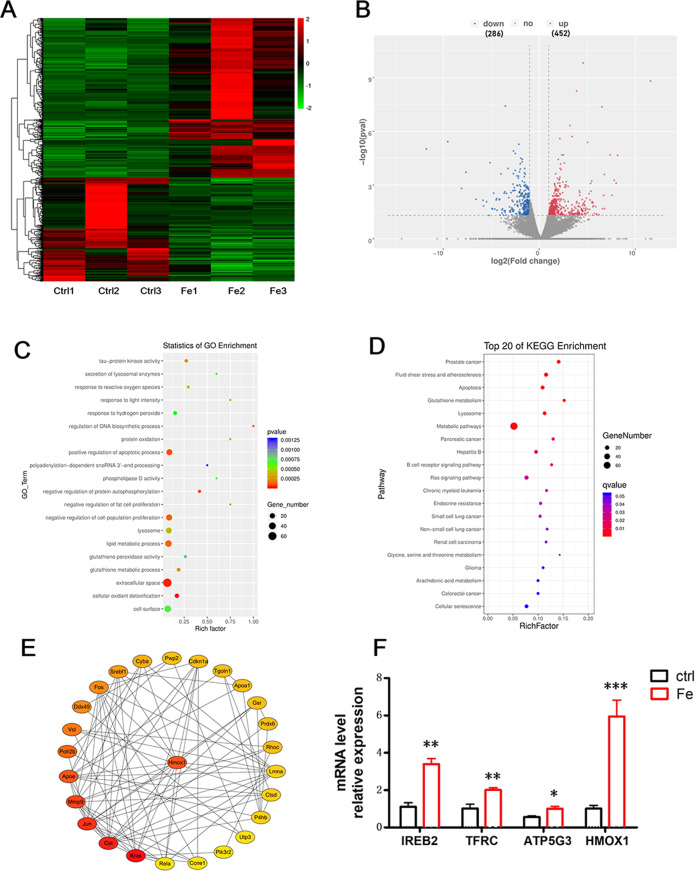
Differential gene expression between control and Fe-treated mouse embryos. **A** Heat map of differentiated expression genes in mouse embryos between control and Fe-treated. Low expression is depicted in green, and high expression is depicted in red. **B** Volcano plot of gene expression (Fe vs. Ctrl; fold change ≥2; *q* value < 0.05), DEGs are represented by red dots (up regulated) and blue dots (down regulated), Non-DEGs are represented by gray dots. The abscissas represent the fold change in genes between the two experimental groups, and the ordinates represent the significance in the fold change in the gene expression. **C** GO analysis of differentially expressed genes in mouse embryos between the control and Fe-treated. **D** KEGG pathway analysis of RNA-seq data between control and Fe-treated mouse embryos. **E** The network of hub genes. The node represent genes, the shade of color indicates weighted degree score, and the black lines represent interaction. **F** The mRNA levels of HMOX1 and ferroptosis marker genes (IREB2, TFRC, and ATP5G3) were measured in Fe-treated mouse embryos and control group. *P < 0.05, **P < 0.01, ***P < 0.001.

To identify the molecular mechanism involved in iron overload-induced ferroptosis in mouse embryos, we used single cell sequencing (Smart-seq) analysis to determine which genes were differentially expressed in the embryos of Fe-treated and control groups. After sequencing, differentially expressed gene (DEG) analysis was performed using read count data obtained from the gene expression level analysis. Genes with the same or similar expression patterns were clustered and visualized by heat maps (Fig. 5A). Volcano plots were generated to visually represent the DEG distribution. A total of 452 genes were significantly upregulated, while 286 genes were downregulated (Fig. 5B). GO analysis revealed that Fe treatment weakened glutathione peroxidase activity and downregulated genes related to lipid metabolic processes, cellular oxidant detoxification and cell population proliferation (Fig. 5C). KEGG analysis showed that the top 20 enrichment items were those related to glutathione metabolism, apoptosis, and antioxidant stress signaling pathways (Fig. 5D).

Hub genes in the coexpression network are a class of genes with high connectivity within a network module and are significantly correlated with biological function [21]. We performed WGCNA to reconstruct the gene regulatory networks (GRNs) and identify the hub genes related to Fe-induced ferroptosis in embryos. The degree-based topological analysis of the network revealed 26 hub genes (Fig. 5E). Among these hub genes, HMOX1 was one of the most significantly upregulated. Real-time PCR confirmed that Fe treatment led to a significant increase in the mRNA levels of *HMOX1* (Fig. 5F). We also tested the expression levels of genes related to ferroptosis and iron metabolism. The mRNA levels of the ferroptosis-related genes ATP synthase F0 complex subunit C3 (*ATP5G3*), iron-responsive element-binding protein 2 (*IREB2*) and transferrin receptor 2 (*TFRC*), which import iron into cells to promote ferroptosis, were upregulated in the Fe-treated group compared with those in the control group (Fig. 5F).

**Fig. 6:**
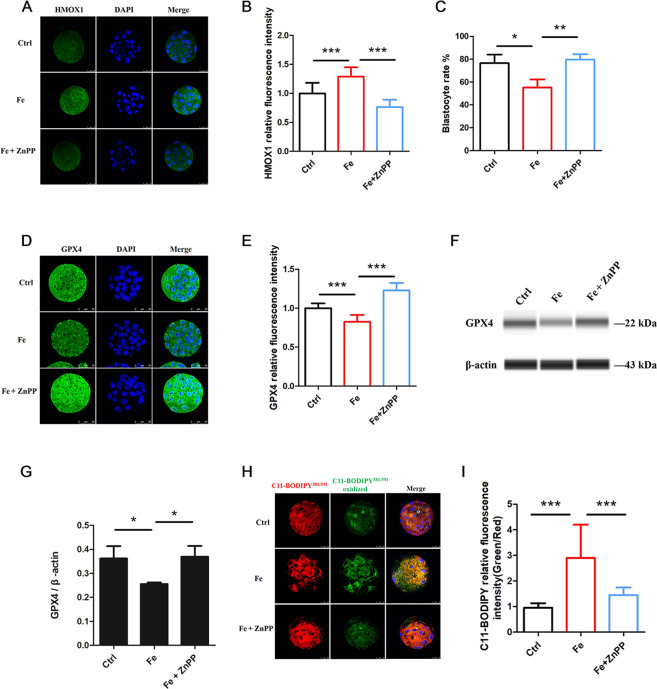
HMOX1 is essential for iron overload induced embryo ferroptosis. **A** Representative immunostaining for HMOX1 in mouse embryos treated with 100 μM Fe and 100 μM Fe +10 μM ZnPP. Nuclei were stained blue with DAPI, the scale bar = 25 μm. **B** Relative fluorescence intensity statistics of HMOX1 in the control (*n* = 14), Fe (*n* = 14) and Fe + ZnPP (*n* = 12). Data are expressed as mean ± SD. ***P < 0.001. **C** The blastocyst rate of mouse embryos cultured with 100 μM Fe and 100 μM Fe + 10 μM ZnPP. *P < 0.05, **P < 0.01. **D** Representative immunostaining for GPX4 in mouse embryos treated with Fe and Fe + ZnPP. Nuclei were stained blue with DAPI, the scale bar = 50 μm. **E** Relative fluorescence intensity statistics of GPX4 in the control (*n* = 16), Fe (*n* = 15) and Fe + ZnPP (*n* = 18). Data are expressed as mean ± SD. ***P < 0.001. **F** Simple western immunoblots showed GPX4 expression in mouse embryos from control, Fe and Fe + ZnPP. β-actin is shown as loading control. **G** Quantification analysis of GPX4 expression in embryos from each treatment, (*n* = 3). Data are expressed as mean ± SD. *P < 0.05. **H** The lipid peroxidation stained by BODIPY 581/591 C11 in mouse embryo cultured with Fe and Fe + ZnPP. Red representing non-oxidized BODIPY 581/591 C11, green representing oxidized BODIPY 581/591 C11, the scale bar = 25 μm. **I** Relative fluorescence intensity statistics of BODIPY 581/591 oxidation (Green/Red) in control (*n* = 24), Fe (*n* = 25), and Fe + ZnPP (*n* = 24). Data are expressed as mean ± SD. ***P < 0.001.

To characterize the functional role of HMOX1 in the pathogenesis of Fe-induced ferroptosis in mouse embryos, we treated Fe group embryos with the competitive HMOX1 inhibitor zinc protoporphyrin IX (ZnPP) which could reduce the expression of HMOX1 (Fig. 6A, B). The results showed that ZnPP significantly increased blastocyst formation (Fig. 6C) and the expression of GPX4 compared with the Fe group (Fig. 6D–G). Fe-induced lipid peroxidation was also blocked by ZnPP (Fig. 6H, I). The above results strongly suggest the important effect of HMOX1 in embryo ferroptosis caused by iron overload.

### Suppression of HMOX1 against iron overload induces ferroptosis by maintaining mitochondrial function

**Fig. 7:**
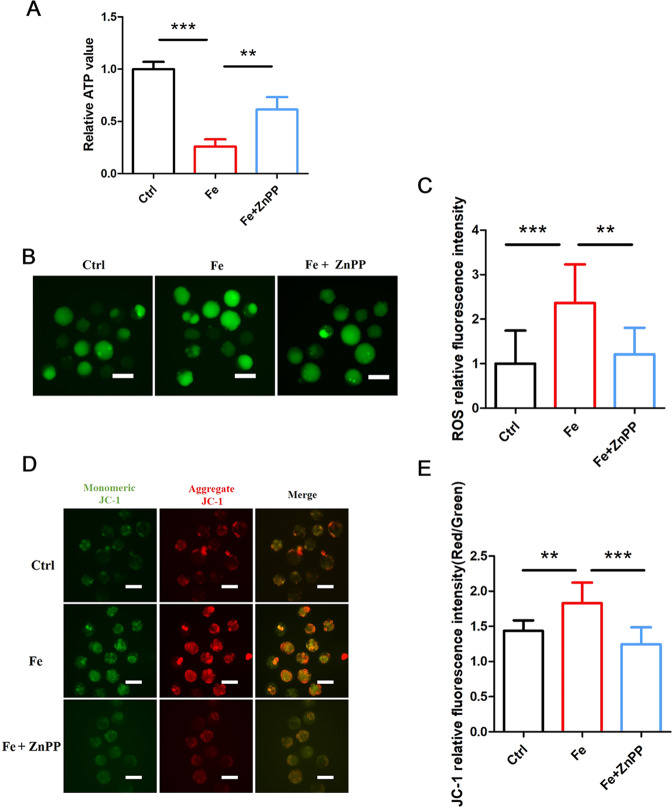
Inhibition of HMOX1 can recuse mitochondrial function. **A** ATP of mouse embryos cultured with 100 μM Fe and 100 μM Fe + 10 μM ZnPP were measured using a bioluminescent assay system. Data are represented as mean ± SD. **P < 0.01, ***P < 0.001. **B** Reactive oxygen species (ROS) detection of mouse embryos cultured with Fe and Fe + ZnPP, the scale bar = 100 μm. **C** Relative fluorescence intensity statistics of ROS in control (*n* = 15), Fe (*n* = 12) and Fe + ZnPP (*n* = 12). Data are expressed as mean ± SD. **P < 0.01, ***P < 0.001. **D** MMP detection of mouse embryos cultured with Fe and Fe + ZnPP by using JC-1 staining. The scale bar = 100 μm. **E** Relative fluorescence intensity statistics of JC-1 (Red/Green) in control (*n* = 12), Fe (*n* = 14) and Fe + ZnPP (*n* = 10). Data are expressed as mean ± SD. **P < 0.01, ***P < 0.001.

To further uncover the roles of HMOX1 in the function of Fe-induced ferroptosis, we next assessed the influence of HMOX1 on mitochondrial function. Similar to previous experimental results, Fe caused a strong deficiency in ATP production in embryos, but this effect was rescued by ZnPP (Fig. 7A). We investigated the total ROS levels in Fe-treated embryos after suppressing HMOX1. The increased oxidative stress-related ROS caused by Fe could also be blocked by ZnPP (Fig. 7B, C). To evaluate the MMP, we also found that the mitochondrial membrane potential hyperpolarization of Fe group embryos could be recovered by ZnPP treatment (Fig. 7D, E), suggesting that ZnPP-mediated inhibition of ferroptosis protects mitochondrial function. Thus, the data on HMOX1 inhibition indicate its essential role in embryo ferroptosis and maintaining mitochondrial function.

## Discussion

Endometriosis has been associated with iron overload in the peritoneal cavity. Our findings confirm that iron and ferritin concentrations are increased in the PF of patients with endometriosis (Fig. 1). The results are consistent with the findings of Van Langendonckt et al. [10] and Lousse et al. [22]. In endometriosis PF, more transferrin is bound by iron, as confirmed by higher transferrin saturation. Transferrin saturation was also higher in the PF of endometriosis patients as Lousse et al. reported [22]. Retrograde menstruation is considered an essential step in the pathogenesis of peritoneal endometriosis, transporting menstrual endometrial tissue through the Fallopian tubes into the peritoneal cavity [23]. Iron overload may originate from the lysis of pelvic erythrocytes [24] and cause significant cytotoxic effects on cells such as endometrial stromal cells [25, 26].

We first focused on iron overload that may cause embryotoxicity in endometriosis. Factors present in PF are related to the mechanisms that cause associated infertility [27, 28], such as an increase in sperm phagocytosis [29]. Illera et al. [30] observed that endometriosis PF had a detrimental effect on embryonic implantation in a mouse model by wrecking uterine receptivity. Alterations in the production of cytokines in the PF, such as IL-6 [6], EGF and IGF-1 [31], contribute to increases in embryotoxicity and induce apoptosis in embryos. Here, we reported that ferroptosis plays an important role in iron overload-induced embryotoxicity (Fig. 2).

To confirm the effect of iron overload on embryos, we treated embryos with Fe and found that the Fe group presented a significantly lower blastocyst rate and induced embryo ferroptosis (Fig. 3). Iron accumulation could lead to excessive ROS production and impaired cell injury [32], which is a finding consistent with our conclusion. Oxidative stress is associated with postovulatory oocyte aging and apoptosis in early embryos [33, 34]. Additionally, ATP and MMP, which represent the function of mitochondria, were abnormal in Fe-treated embryos in our study, demonstrating that mitochondria play a central role in embryo ferroptosis caused by iron overload (Fig. 4). Presumably, MMP hyperpolarization reflects an increase in mitochondrial electron transport chain activity and subsequent lipid ROS generation, explaining the role of mitochondria in ferroptosis [17]. Therefore, the harmful effect on mitochondria caused by iron overload may be a crucial pathogenic factor in the development of infertility in endometriosis.

Another important finding in our study was that HMOX1 plays a large role in the response to iron overload-induced embryonic ferroptosis (Figs. 5 and 6), and this process may be related to damage to mitochondrial function (Fig. 7). HMOX1 is considered as an anti-oxidative and anti-inflammatory gene that protects cells from ROS assault. However, increasing evidence indicates that HMOX1 overexpression has pro-oxidant effects and induces ferroptosis by augmenting iron accumulation and lipid peroxidation [35–37]. Fang et al. reported that the inducible heme oxygenase Hmox1 is upregulated by the nuclear translocation of Nrf2 in DOX-induced cardiac ferroptosis, and mitochondrial lipid peroxidation due to mitochondrial iron accumulation plays a key role in this process [38]. Suppression of HMOX1 function, resulted in decreased iron levels and subsequent Fenton chemistry, likely indicating that HMOX1 is the actual downstream link in response to PRDX6 silencing-enhanced ferroptosis [39]. Determing the pathway of HMOX1 involved in embryo ferroptosis in endometriosis requires further investigation.

In conclusion, our data suggest that iron overload in the peritoneal fluid of endometriosis may cause embryotoxicity and induce ferroptosis. HMOX1 may play an important role in this process. Our research may provide new insight into the mechanism of endometriosis infertility. More work is needed to understand the role of HMOX1 to facilitate the identification of optimal targets for future endometriosis infertility treatment efforts.

## Materials and methods

### Patients and peritoneal fluid collection

This study was approved by the Ethics Committee of Zhejiang Provincial People’s Hospital, Hangzhou Medical College (approval number 2019KY189), and all patients provided written informed consent. Seventy-two patients seen for infertility at the Reproductive Medicine Center of Zhejiang Provincial People’s Hospital were included. The exclusion criteria were receiving hormonal therapy during the last 3 months, pregnancy or a history of other intra-abdominal surgery. The patients were divided into two groups. The control group (*n* = 31; 33.74 ± 9.04 years) had no endometriotic lesions detected by laparoscopy, and women with endometriosis (*n* = 41; 32.44 ± 5.78 years) had visible peritoneal endometriotic lesions found by laparoscopy. The morphologic characteristics of endometriosis were confirmed by the presence of both glandular epithelium and stroma in all peritoneal lesions [40, 41].

PF samples were aspirated using a sterile syringe at the beginning of the laparoscopic surgery after trocar insertion and before any significant intra-abdominal manipulation. The samples were immediately centrifuged at 3000 × *g* for 10 min at 4 °C to remove the cellular contents. Next, the supernatant was collected, passed through a 0.22 μm filter (Millipore, USA) and stored at –80 °C. Specimens with blood contamination or hemolysis were excluded.

### Detection of iron metabolism

Iron metabolism-related data were measured. The PF iron concentration and unsaturated iron binding capacity (UIBC) were determined using a colorimetric kit by the Ferene method (iron and total binding capacity (TIBC); Leadman, China) and measured using the Beckman–Coulter AU 5800 system (Beckman Coulter, USA). The ferritin concentration was measured using the immunoturbidimetric method (Abbott Laboratories, USA) by ARCHITECT-i2000 (Abbott Laboratories, USA). Transferrin was determined by the turbidimetric method using the Transferrin assay kit (Beckman Coulter Inc, USA) and the Immage® 800 Rate Nephelometer (Beckman Coulter Inc, USA). Transferrin saturation (TSAT) was calculated based on the iron concentration and TIBC (TSAT = Fe/TIBC × 100).

### Animal handling and embryo collection

C57BL/6J mice were purchased from Shanghai SLAC Animal Laboratory. The mice were housed in cages maintained under a constant 12 h light/12 h dark cycle at 21–23 °C with unlimited access to standard chow and water in a specific pathogen-free (SPF) vivarium at Zhejiang Provincial People’s Hospital. Female mice aged 4–6 weeks were superovulated by intraperitoneal injection with 10 IU of pregnant-mare serum gonadotropin, followed by treatment with 10 IU of human chorionic gonadotropin (HCG) 48 h later, and then caged immediately with proven male breeders at a 1:1 ratio. Successfully mated females with vaginal plugs checked the following morning were euthanized 36 h after HCG treatment to collect 2-cell embryos. The oviducts from donor mice were transferred to a clean dish with MOPS medium (Vitrolife, Sweden). Two-cell stage embryos were released from the oviduct under a thermal platform stereomicroscope and washed twice with MPOS containing 10% v/v serum substitute (Irvine Scientific, USA).

All experimental protocols concerning the use and care of mice in this study were reviewed and approved by the Institutional Animal Ethics Committee of Hangzhou Medical College (approval number 20190194).

### Embryo culture

Embryo culture was performed in 50–60 μL of medium covered by mineral oil at 37 °C and 6% CO_2_. Two-cell embryos were transferred into G1 plus medium (Vitrolife, Sweden) supplemented with a 7% solution of the PF of patients with or without endometriosis (endometriosis or control groups, respectively) or containing the following: 1) Fe group, iron (ferrous sulfate powder, Sigma, USA) at concentrations of 100 μM; 2) Fer-1 group, iron at 100 μM and Fer-1(2, 10, 50 μM); 3) ZnPP group, iron at 100 μM and ZnPP at 10 μM; 4) medium only as a blank control. The embryos were then washed and cultured in G2 plus medium (Vitrolife, Sweden) for another 48 h to reach the blastocyst stage. The scoring and collection of embryos at various developmental stages were based on the embryo morphology approximately as follows, following HCG injection: 2-cell, 36 h (E1.5, in vitro culture initiated); cleavage, 60 h (E2.5, in vitro cultured for 24 h); morula, 72 h (E3, in vitro cultured for 36 h); and blastocyst, 96 h (E4, in vitro cultured for 60 h).

### Quantitation of ATP production

The relative ATP level was measured using the ENLITEN® ATP Assay System Bioluminescence Detection Kit (Promega, USA) according to the manufacturer’s instructions. Embryos with or without treatment were lysed in cell lysis buffer. The ATP content of the embryo was then detected in a 96-well white opaque plate containing luciferase, luciferin, and buffer by bioluminescence on a multimode microplate reader (TECAN, Swit).

### Detection of the mitochondrial membrane potential

The mitochondrial membrane potential (MMP) in embryos was measured by staining with 5,6,6-dichloro-1,1,3,3-tetraethyl-imidacarbocyanine iodide (JC-1) according to the manufacturer’s instructions (Beyotime, China). The embryos were incubated in MOPS medium containing 1 μM JC-1 for 15 min at 37 °C in the dark. After incubation, the embryos were washed twice with PBS and observed under an epifluorescence microscope (Nikon ECLIPSE Ti, Japan) equipped with a laser source at excitation/emission wavelengths of 488/510 nm (to visualize green JC-1 monomers) or 561/590 nm (to visualize red JC-1 aggregates). Image J software was used to quantitatively analyze the fluorescence intensity of acquired images with JC-1 staining. The MMP was calculated as the ratio of the fluorescence intensity at 590/510 nm.

### Assessment of oxidative stress

The intracellular level of ROS, as an indicator of oxidative stress in embryos, was measured by 2′,7′-dichlorofluorescein diacetate (DCHFDA) according to the manufacturer’s instructions (Solarbio, China). Briefly, the embryos were incubated in G1 medium containing 10 μM DCHFDA at 37 °C for 15 min in the dark. After washing three times with PBS containing 1 mg/mL polyvinylpyrrolidone (PBS-PVP), the embryos were fixed onto glass slides and observed immediately under an epifluorescence microscope (Nikon ECLIPSE Ti, Japan) at an excitation wavelength of 490 nm and an emission wavelength of 525 nm. Image J software was used to analyze the fluorescence intensity in embryos.

### Immunofluorescence (IF) staining

Ferroptosis induction in the embryos was determined by GPX4 expression using IF staining. Briefly, after washing three times with PBS, embryos were fixed in 4% paraformaldehyde on ice for 15 min and incubated in 0.1% Triton X-100 permeabilization solution at room temperature (RT) for another 15 min. Next, the embryos were incubated in blocking solution for 30 min and then in primary antibody (1:200) against GPX4 (Abcam; ab125066), HMOX-1 (Proteintech; 66743) for another 60 min at RT. After extensive washing and further blocking, the embryos were incubated in blocking solution with 1:500 goat anti-rabbit IgG H&L Alexa Fluor® 488 (Abcam; ab150077) for 60 min at RT and finally mounted onto slides for counterstaining with DAPI in mounting medium under a cover slip. Fluorescence was detected and imaged using a confocal laser-scanning microscope (Leica SP8, Germany). The intensity of the fluorescent signal in the embryos was analyzed using Image J software.

### Detection of lipid peroxidation

After treatment, the embryos were stained with 2 μM C11-BODIPY ^581/591^ (Invitrogen, D3861) for 20 min at 37 °C in MOPS containing 10% SSS. The embryos were then collected and washed with PBS. Lipid peroxidation was analyzed by the detection of a fluorescence shift from green to red using a confocal laser-scanning microscope (Leica SP8, Germany),with excitation at 488 and 565 nm and emission recording at 505–550 nm (green) and above 580 nm (red). The intensity of the fluorescent signal was analyzed using Image J software.

### Automated capillary-based simple western immunoblots

Before blotting, the protein was quantified using the bicinchoninic acid (BCA) method. Simple western immunoblotting was performed on a Simple Wes System (ProteinSimple, USA) using a Size Separation Master Kit with Split Buffer (12–230 kDa) according to the manufacturer’s standard instruction and using anti-GPX4 (Abcam; ab125066) and anti-β-actin (Abcam; ab8227) antibodies. Compass software (version 4.0.0, ProteinSimple) was used to program the Simple Wes and for presentation (and quantification) of the western immunoblots. Output data were displayed from the software calculated average of seven exposures (5–480 s).

### Real-time PCR

Total RNA was extracted using the miRNeasy Kit QIAGEN (Qiagen, USA). One microgram of total RNA was used to synthesize cDNA (Takara, Shiga, Japan). For PCR amplification, a 20 mL reaction volume included 10 mL of 2× SYBR Premix Ex Taq mixture (Takara, Japan), 0.2 mmol/L of each primer, 2 mL of twofold diluted cDNA and sterile distilled water according to the manufacturer’s protocols. The cycle threshold (CT) values were collected and normalized to the housekeeping gene β-actin. The 2^−△△CT^ was calculated to estimate the relative mRNA levels of each target gene. The primer sequences of the target gene are shown in Table 2.

**Table 2:** The primer sequences of target gene.

Gene	Sequence (5′ to 3′)
m-IREB2-F	TTCTGCCTTACTCAATACGGGT
m-IREB2-R	AGGGCACTTCAACATTGCTCT
m-TFRC-F	GTTTCTGCCAGCCCCTTATTAT
m-TFRC-R	GCAAGGAAAGGATATGCAGCA
m-ATP5G3-F	TCTGCATCAGTGTTATCTCGGC
m-ATP5G3-R	CACCAGAACCAGCAACTCCTA
m-HMOX1-F	AAGCCGAGAATGCTGAGTTCA
m-HMOX1-R	GCCGTGTAGATATGGTACAAGGA

### RNA extraction and sequencing

Total RNA was extracted using the miRNeasy Kit QIAGEN (Qiagen, USA) according to the manufacturer’s instructions. The RNA quantity and purity were determined using the Bioanalyzer 2100 and RNA 6000 Nano LabChip Kit (Agilent, USA). The mRNAs were enriched using a modified oligo(dT) primer (the SMART CDS Primer) attached to magnetic beads (Invitrogen, USA) and then fragmented into small pieces. These cleaved RNA fragments were reverse transcribed to create the cDNA library using an mRNASeq sample preparation kit (Illumina, USA), and the library was sequenced by the Illumina NovaSeq™ 6000 (LC Sciences, USA).

### RNA-seq data analysis

After sequencing and following the removal of low-quality reads that contained adapter contamination, low-quality bases, and undetermined bases were removed, and the sequenced reads were aligned to the mouse genome using HISAT2. The calculation of mRNA fragments per kilobase of transcript per million mapped reads (FPKM) in each sample was performed using StringTie by summing the FPKMs. The differentially expressed mRNAs and genes were selected using log2 (fold change) >1 or log2 (fold change) <−1 with statistical significance (*P* < 0.05) by Ballgown [42].

Gene ontology (GO) term enrichment is a bioinformatics tool widely used to interpret gene sets with a set of predefined terms to better understand the underlying biological processes of some genes. The selected differentially expressed genes (DEGs) were subjected to GO enrichment; 134 GO terms with *P* < 0.01 were considered significantly enriched, from which we selected 20 terms to explain our results above. Finally, the DEGs were enriched by the Kyoto Encyclopedia of Genes and Genomes (KEGG) pathways. We used WebGestalt (WEB-based Gene SeT AnaLysis Toolkit) to analyze and choose a cut-off based on the *P*-value, and all pathways with a *P*-value less than 0.05 were included in the subsequent analysis. We also selected 20 terms to explain our results above.

### Identification of Hub genes

We used the CytoHubba plug-in in Cytoscape to perform weighted gene coexpression network analysis (WGCNA) and identify hub genes. The degree of connectivity of each gene was determined as the sum of the edge attributes of genes connected to it. The higher the connectivity is, the stronger the biological function of the gene. We selected the top 26 hub genes with a degree >15 and constructed the zero-order network.

### Statistical analysis

All statistical analyses were performed using GraphPad Prism software and data derived from at least three different batches of pooled embryos as biological replicates. Categorical data were checked for the normality of distribution (Kolmogorov–Smirnov test) and homogeneity of variance (Brown–Forsythe test), and means ± standard deviation (SD) were compared using one-way analysis of variance (ANOVA) with Tukey’s multiple comparisons tests for post hoc analysis. The data did not have equal variance even after log transformation analyzed by the nonparametric Kruskal–Wallis test instead of ANOVA. Differences in values were considered significant if *P* < 0.05.

## Supplementary information

confirm the final author list

auther contribution

## Data Availability

The data underlying this article will be shared on reasonable request to the corresponding author.
