# Supplementary material for: Iron overload in endometriosis peritoneal fluid induces early embryo ferroptosis mediated by HMOX1
Source: Cell Death Discov. 2021 Nov 15;7:355. doi: 10.1038/s41420-021-00751-2 (PMC8593044; doi:10.1038/s41420-021-00751-2)

All co-authors agree to the changes of the author list.

Shishi Li


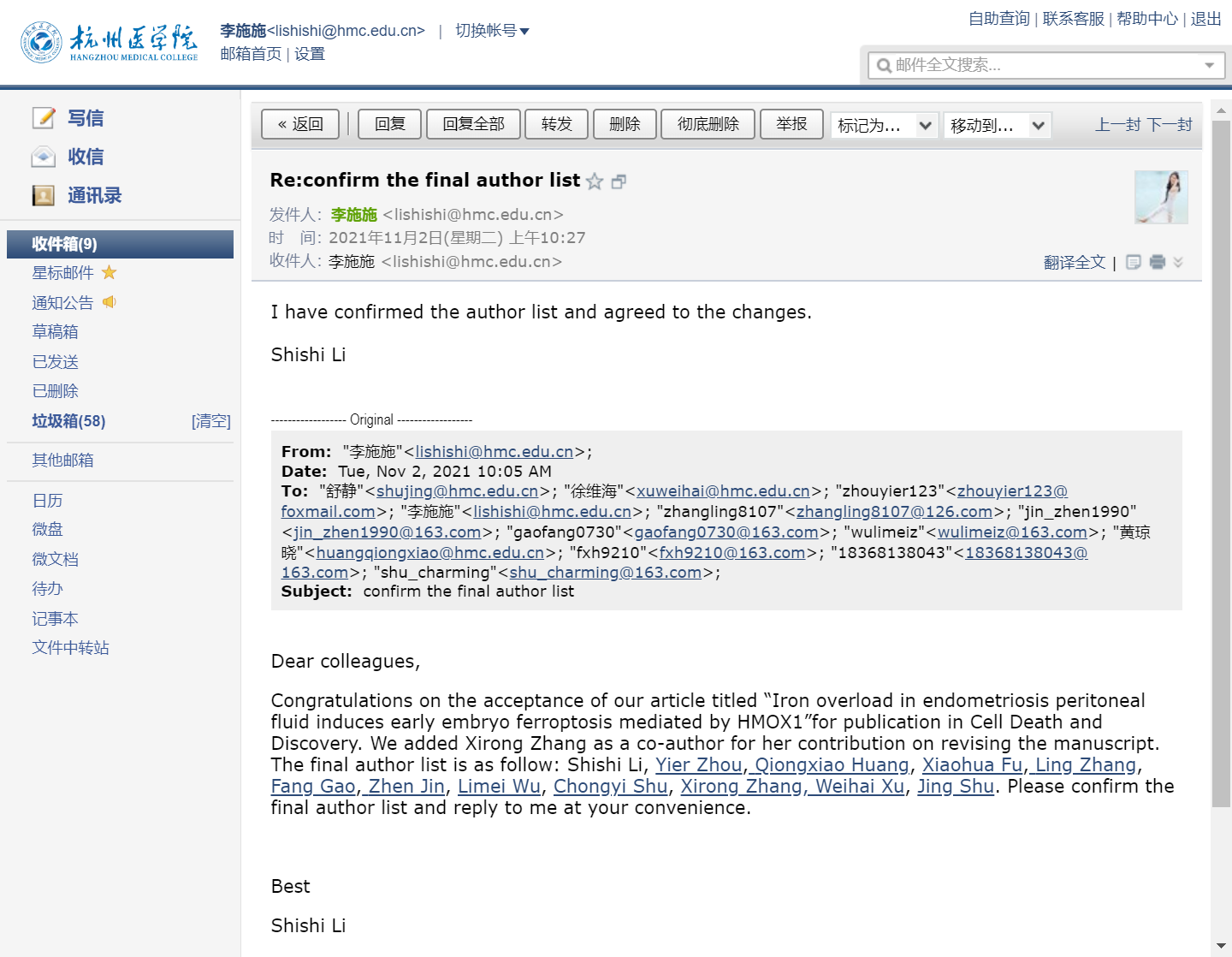


Yier Zhou


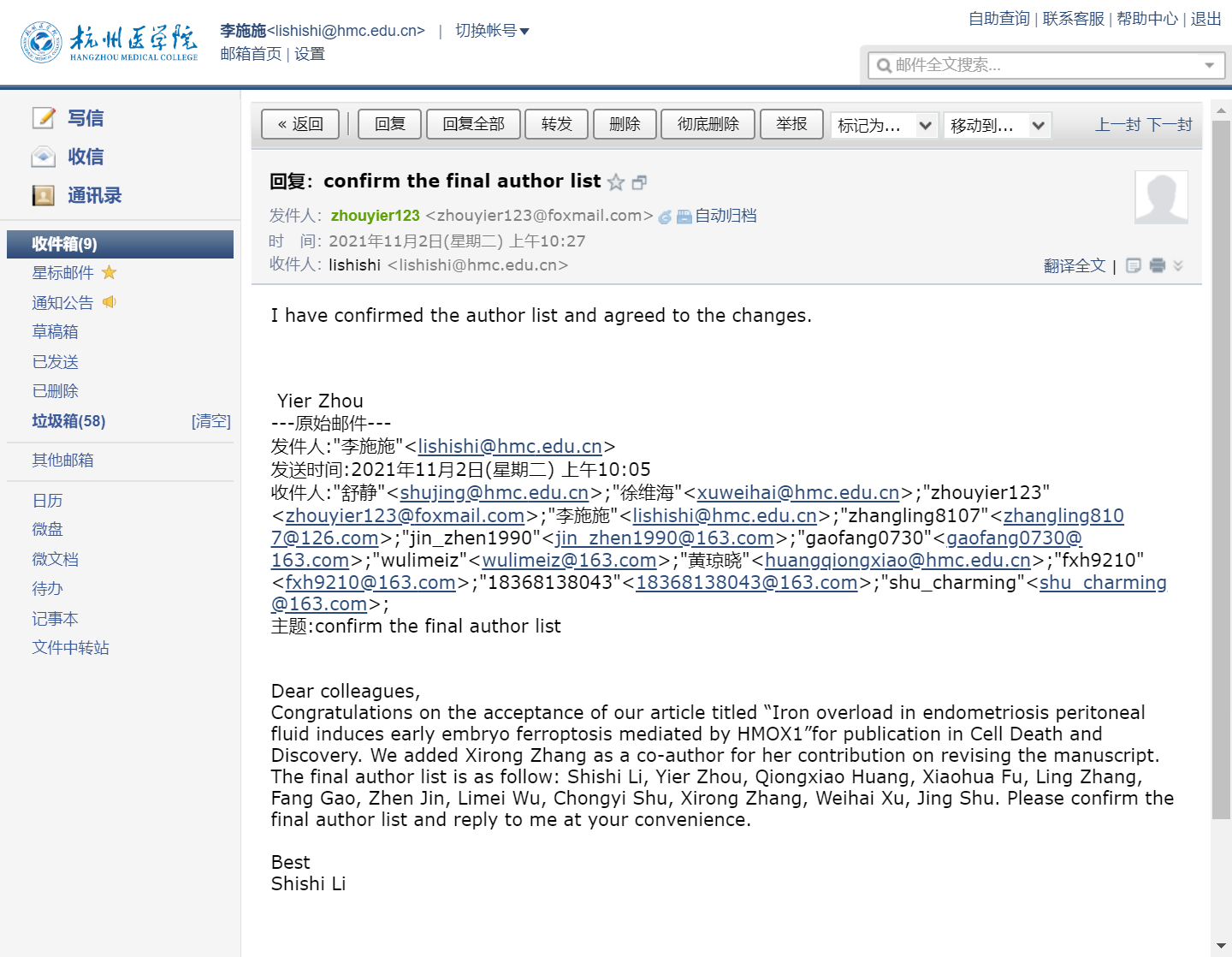


Qiongxiao Huang

qiong


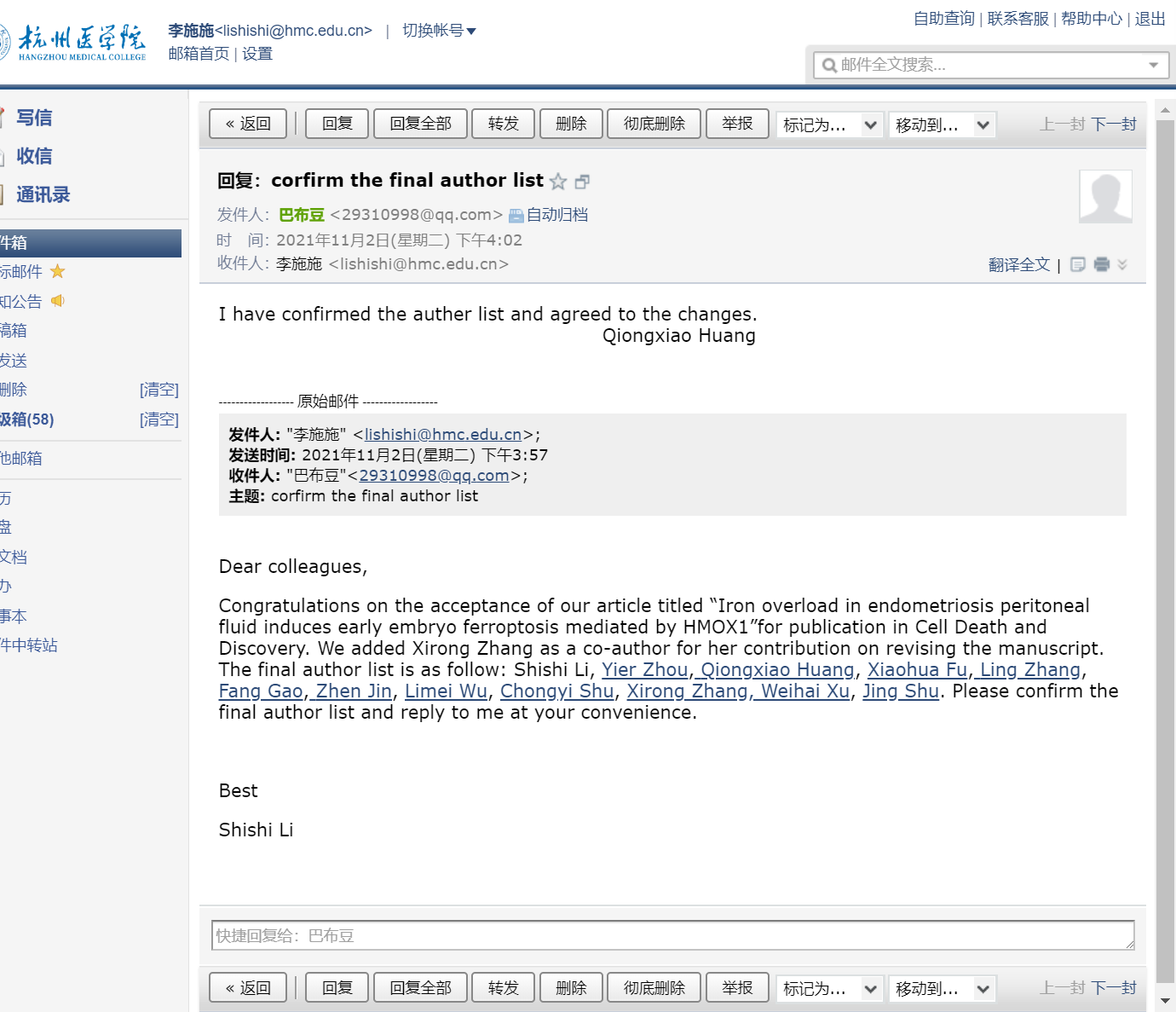


Xiaohua Fu


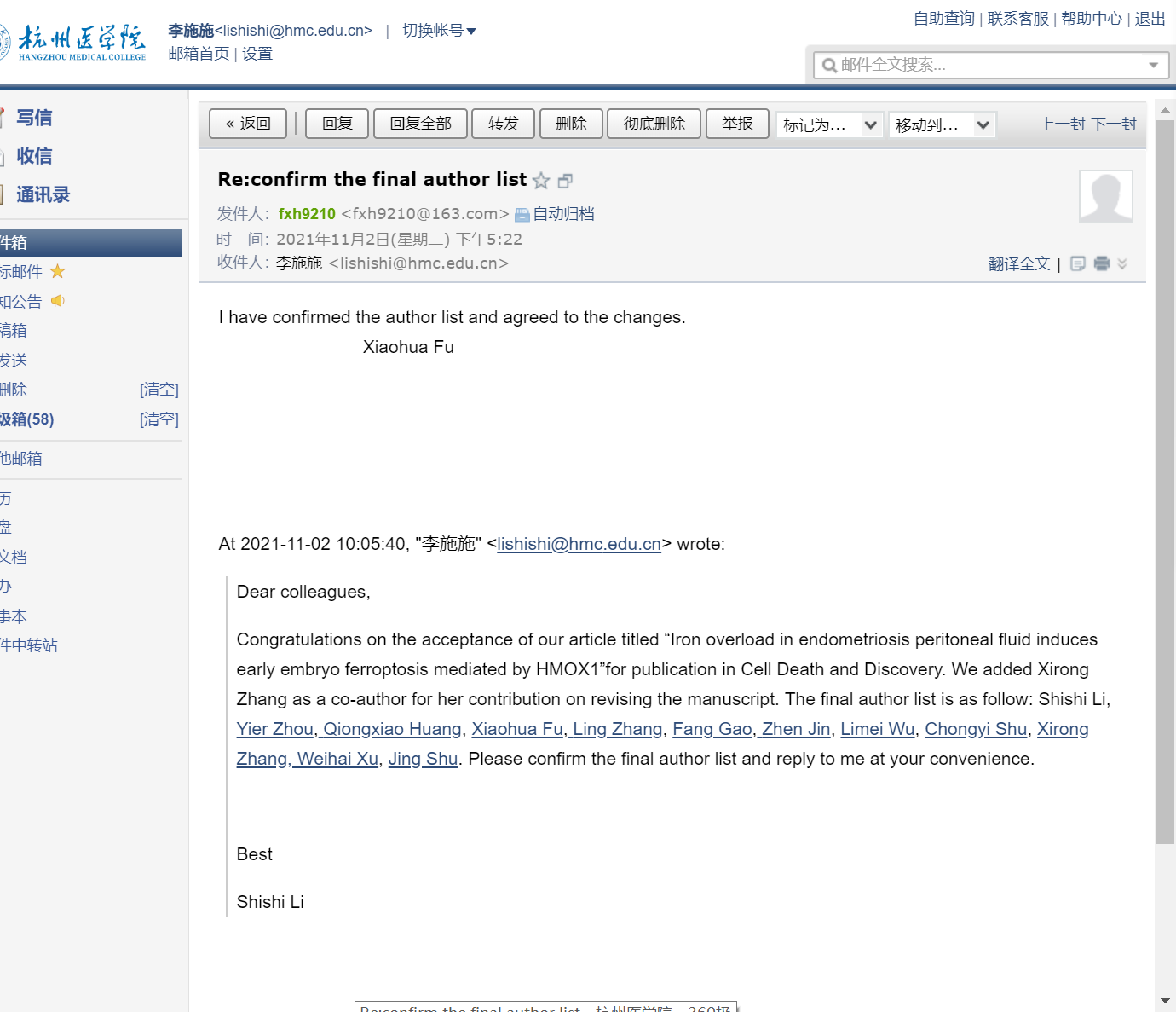


Ling Zhang


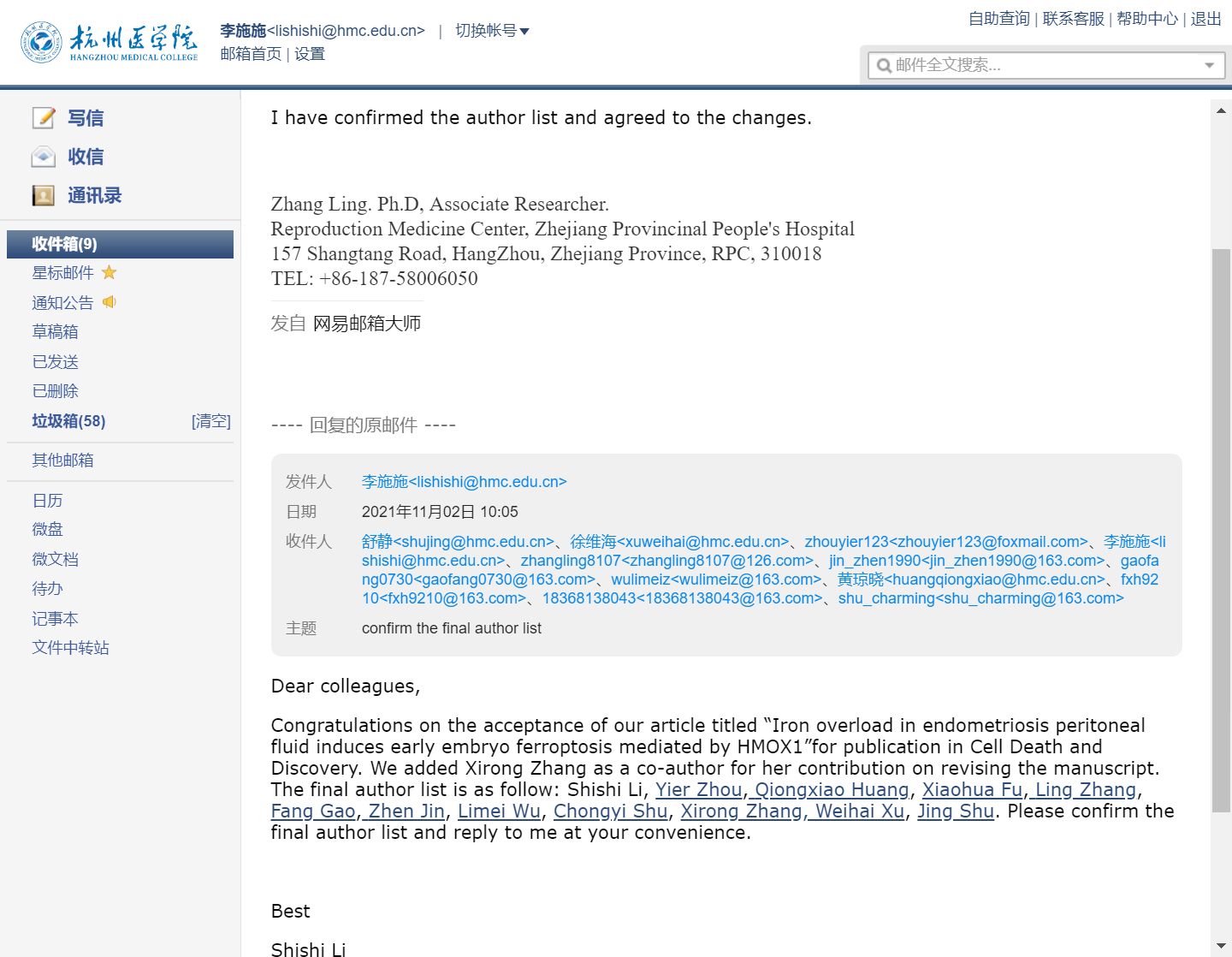


Fang Gao


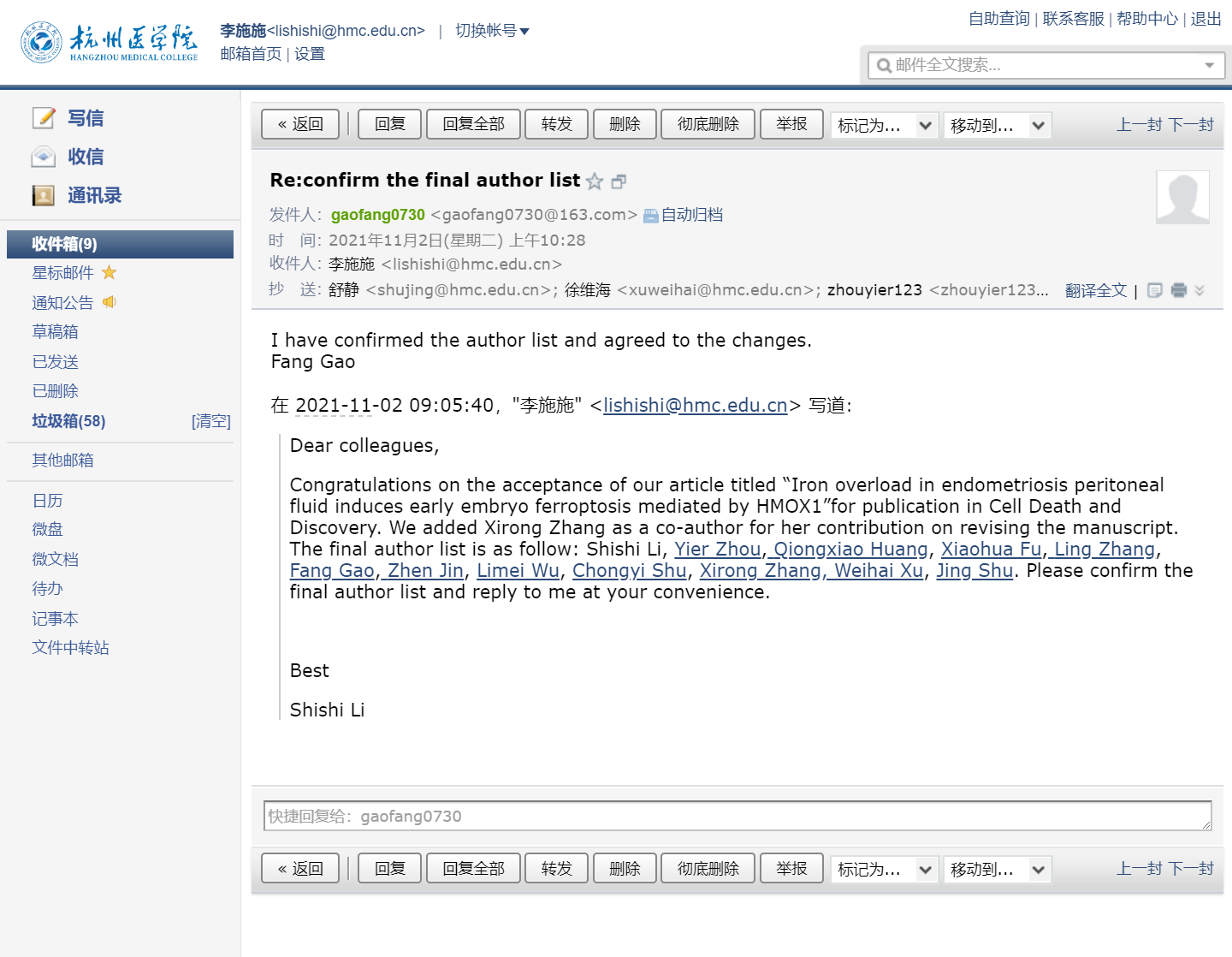


Zhen Jin


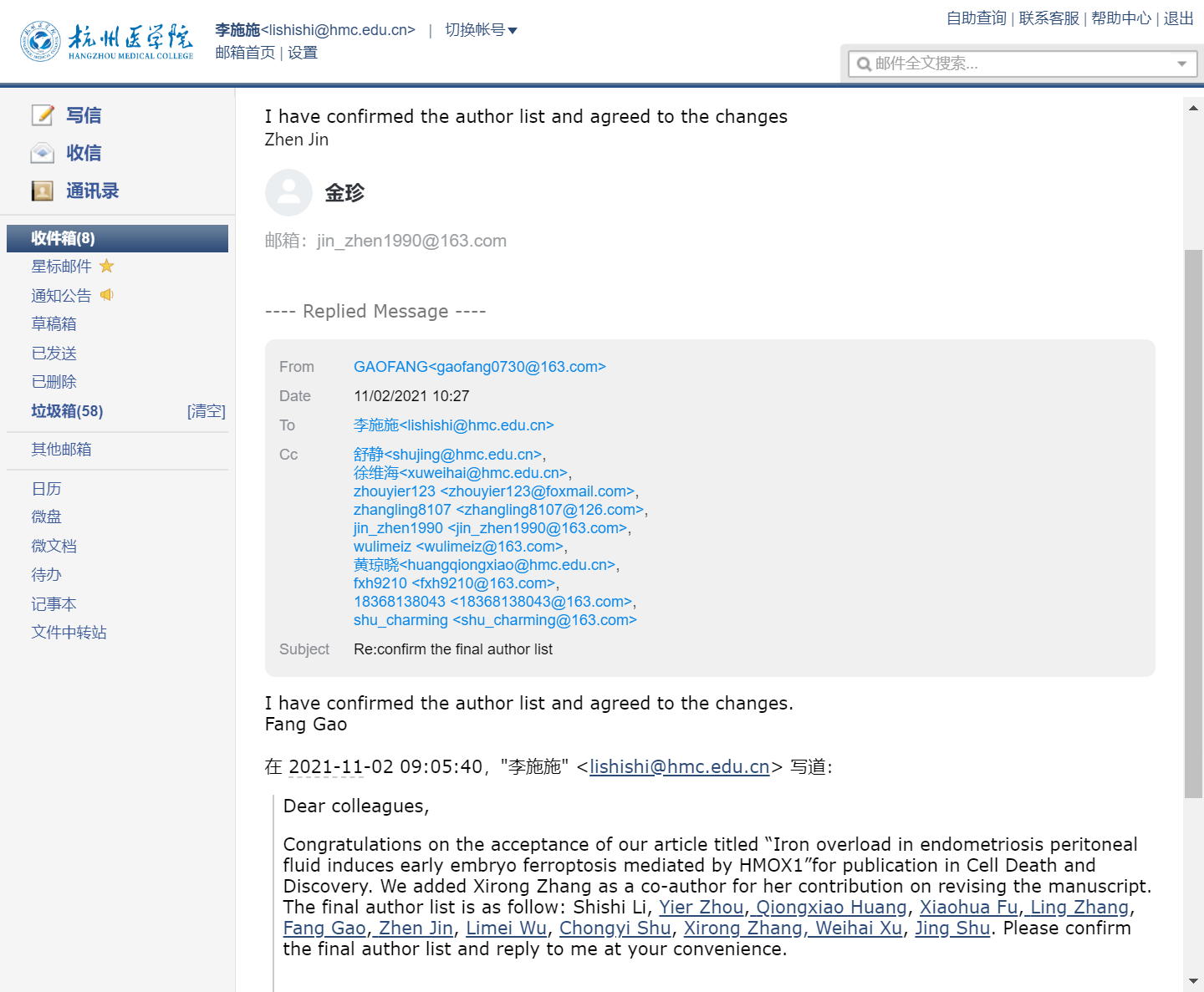


Limei Wu


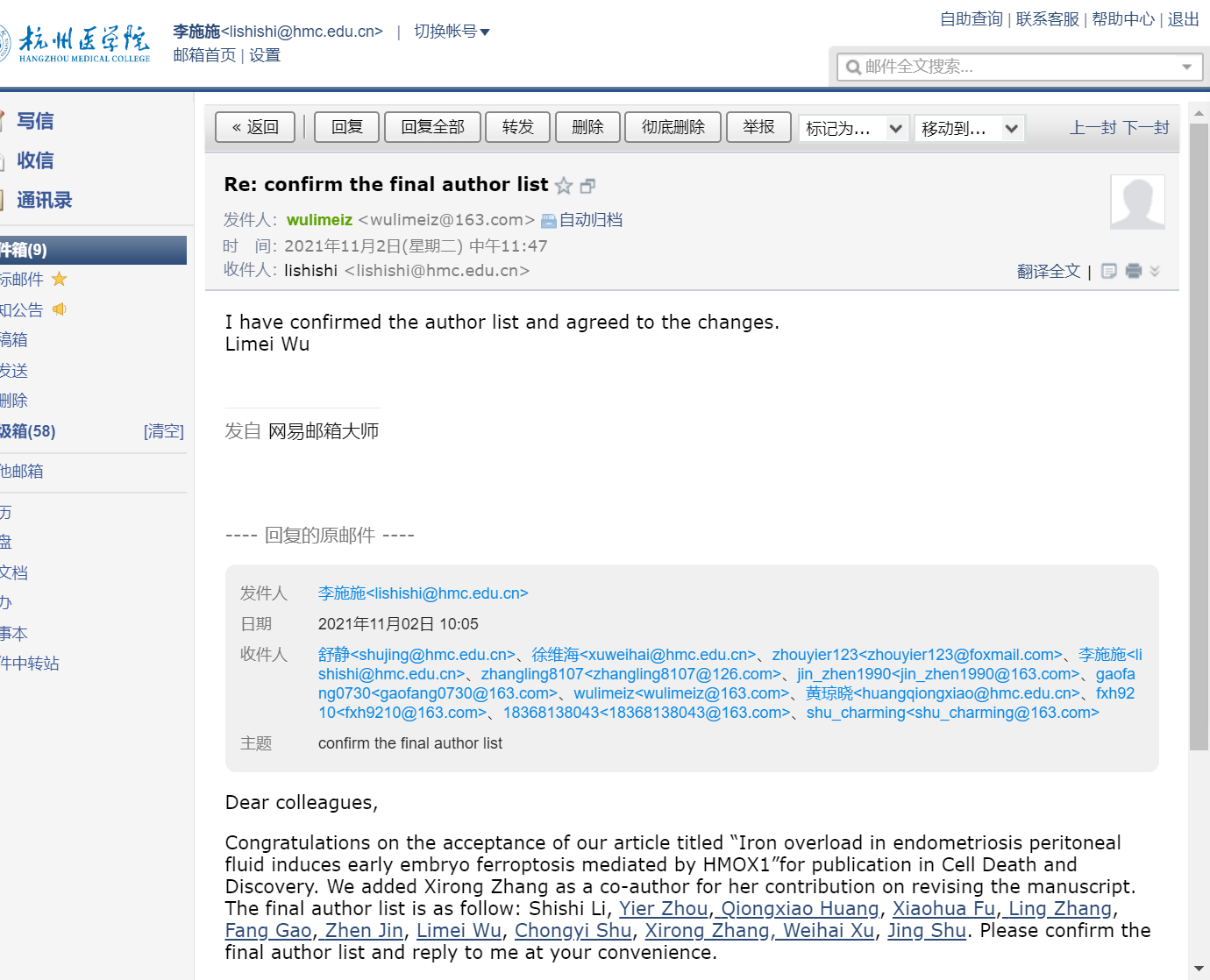


Chongyi Shu


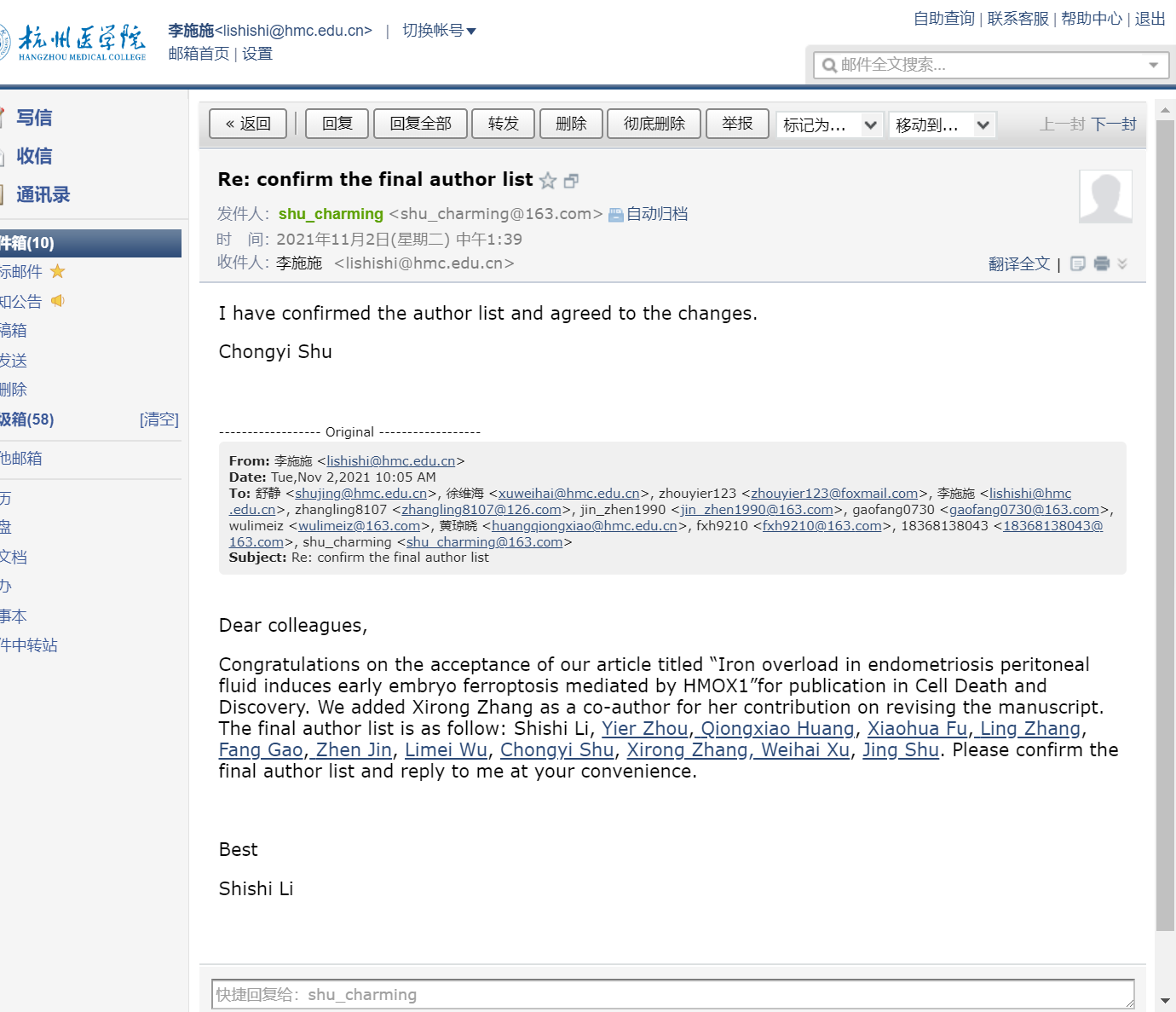


Xirong Zhang


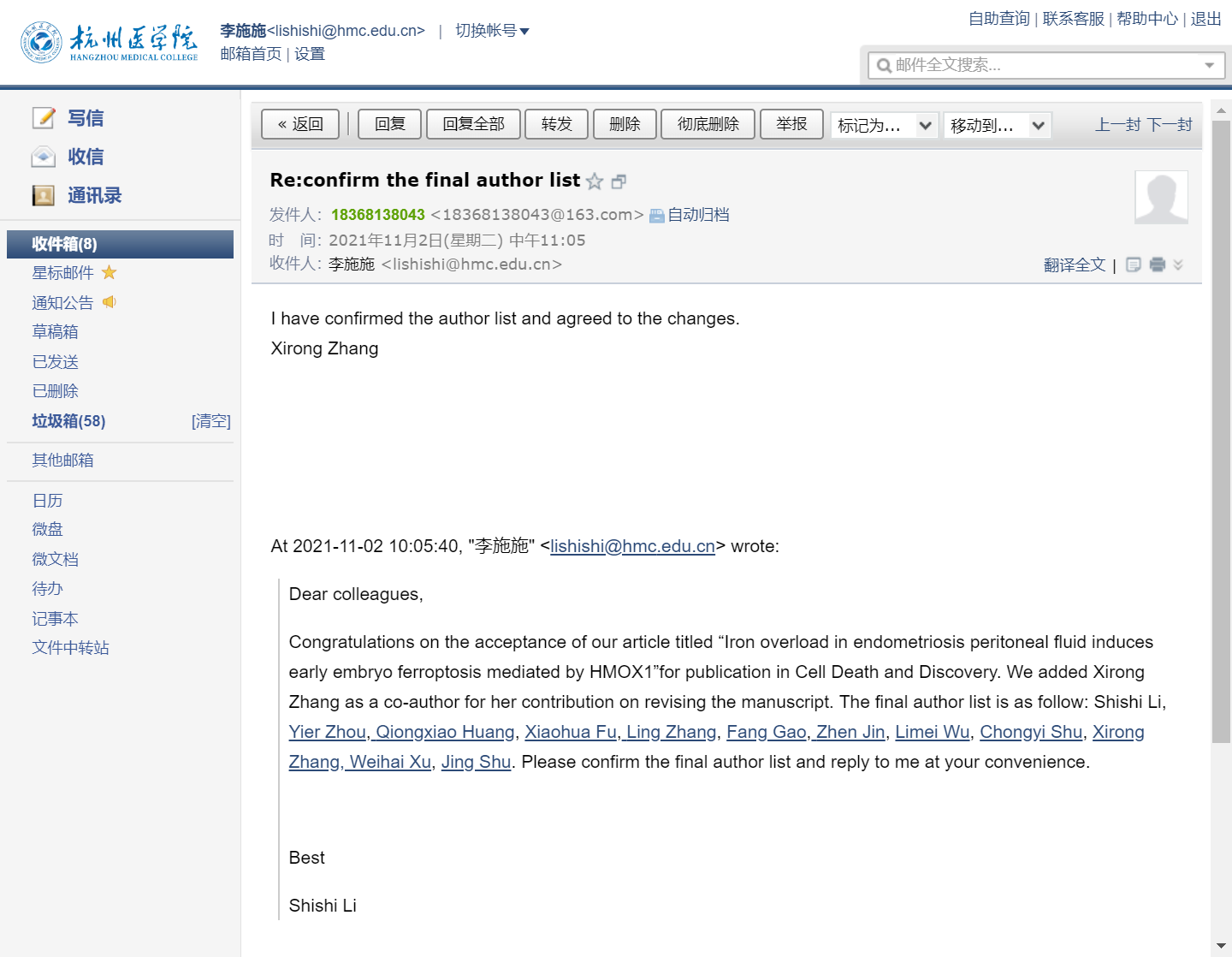


Weihai Xu


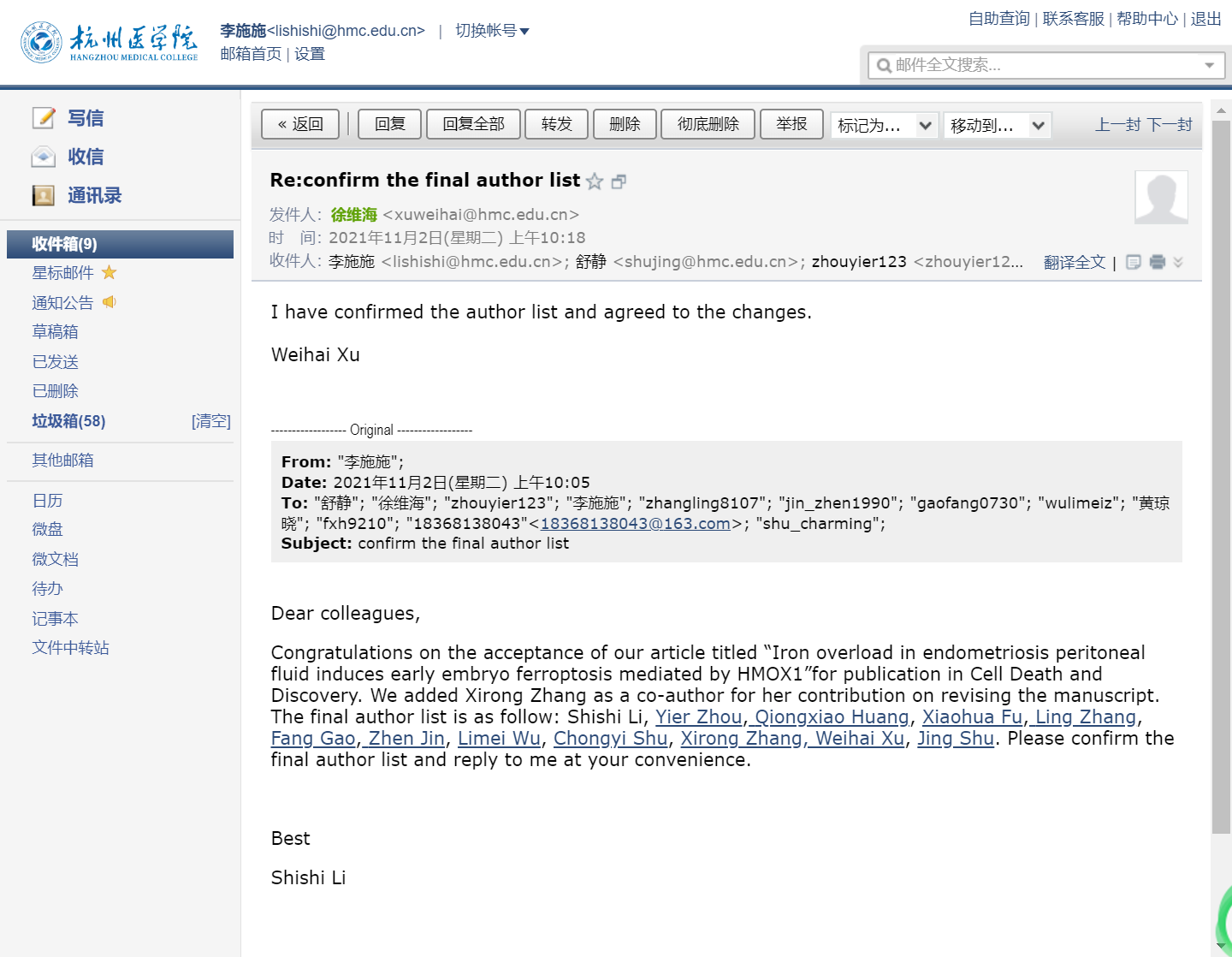


Jing Shu


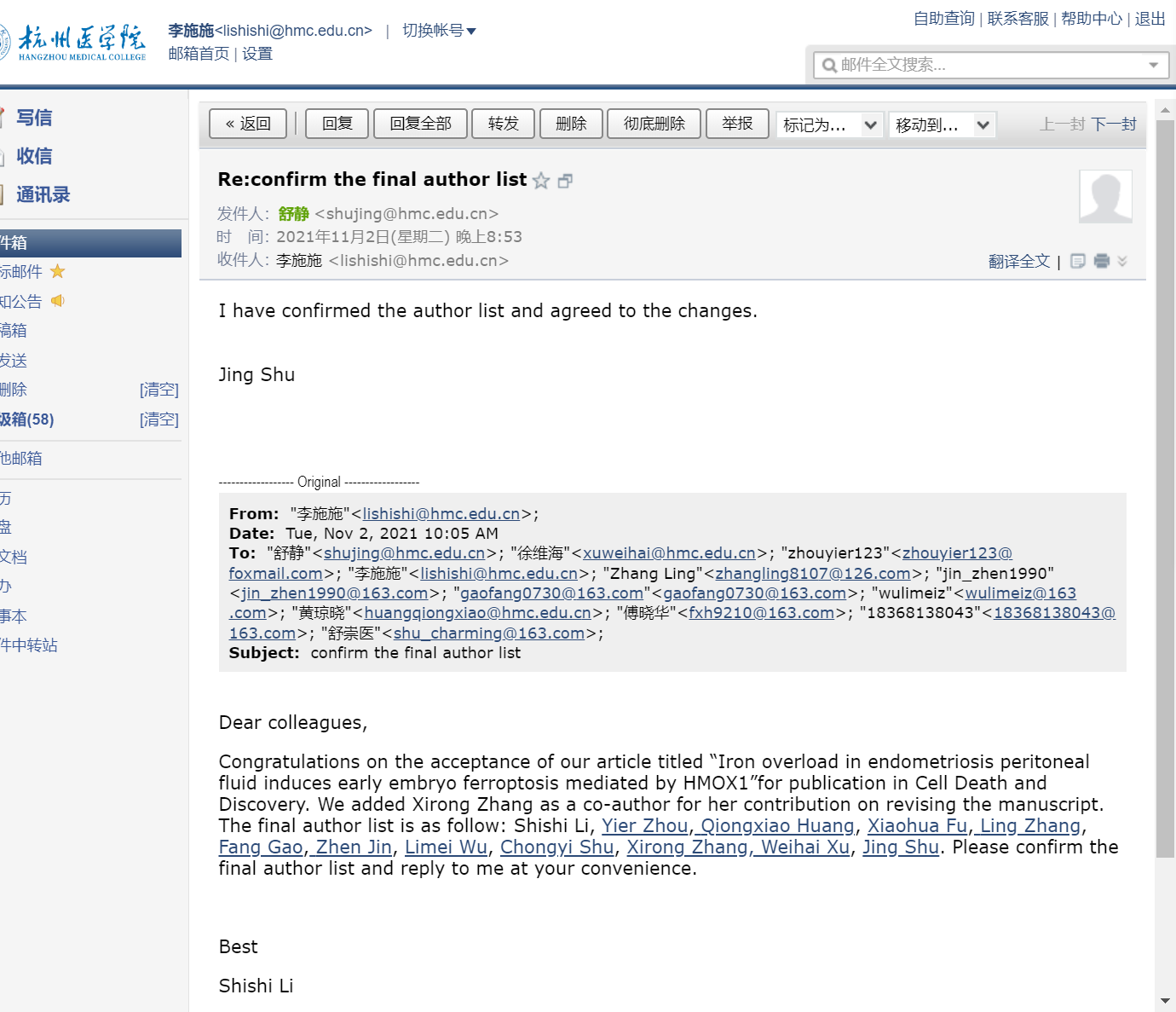

Supplement: Supplementary file 1 — confirm the final author list [file 41420_2021_751_MOESM1_ESM.docx]
