# Supplementary material for: Iron overload in endometriosis peritoneal fluid induces early embryo ferroptosis mediated by HMOX1
Source: Cell Death Discov. 2021 Nov 15;7:355. doi: 10.1038/s41420-021-00751-2 (PMC8593044; doi:10.1038/s41420-021-00751-2)
Supplement: Supplementary file 2 — auther contribution [file 41420_2021_751_MOESM2_ESM.pdf]

DECLARATION OF CONTRIBUTIONS TO ARTICLE

ADMC

Manuscript Number: CDDISCOVERY-21-2148

Journal Name: *Cell Death Discovery* (the 'Journal')

Proposed Title of the Contribution: Iron overload in endometriosis peritoneal fluid induces early embryo ferroptosis mediated by HMOX1 (the 'Contribution)

Author(s): Shishi Li, Yier Zhou, Qiongxiao Huang, Xiaohua Fu, Ling Zhang, Fang Gao, Zhen Jin, Limei Wu, Chongyi Shu,Xirong Zhang, Weihai Xu, Jing Shu (the 'Authors')

For all *CDDiscovery* articles, each person named as an author in the published version must be able to show he or she has contributed substantially to the article.

Authorship credit should be based on 1) substantial contributions to conception and design, acquisition of data, or analysis and interpretation of data; 2) drafting the article or revising it critically for important intellectual content; and 3) final approval of the version to be published. Authors should meet conditions 1, 2 and 3.

Any person who cannot be shown to have made a substantial contribution to the article cannot be listed as an author in the final version. The name of any person who is deemed to have made a minor contribution can, however, appear in the Acknowledgments section of the article.

Please complete the table below to indicate the contributions of all named authors to the manuscript.

Author Full Name:	Specification of Contribution to the Manuscript:
Jing Shu	conceived the project, designed the experiments and edited the manuscript.
Weihai Xu	conceived the project, designed the experiments and edited the manuscript.
Shishi Li	performed the majority of the experiments, interpreted the data and drafted the manuscript.
Yier Zhou	performed the majority of the experiments, interpreted the data and drafted the manuscript.
Qiongxiao Huang	collected patient's peritoneal fluid and detected of iron metabolish.
Xiaohua Fu	collected patient's peritoneal fluid and detected of iron metabolish.
Ling Zhang	contributed to data analysis, and critical discussion.
Fang Gao	carried out the experiments,and critical discussion.
Zhen Jin	carried out the experiments, and critical discussion.
Limei Wu	carried out the experiments, and critical discussion.
Chongyi Shu	carried out the experiments, contributed to data analysis.
Xirong Zhang	carried out the experiments, contributed to data analysis.

Please complete the table below to indicate the contributions of all named authors to the figures.

Figure 1:

Shishi Li, Yier Zhou, Qiongxiao Huang, Xiaohua Fu, Weihai Xu, Jing Shu

Figure 2:

Shishi Li, Yier Zhou, Weihai Xu, Jing Shu,Fang Gao

Figure 3:

Shishi Li, Yier Zhou, Weihai Xu, Jing Shu,Zhen Jin,Xirong Zhang

Figure 4:

Shishi Li, Yier Zhou, Weihai Xu, Jing Shu,Ling Zhang

Figure 5:

Shishi Li, Yier Zhou, Weihai Xu, Jing Shu,Ling Zhang,Limei Wu

Figure 6:

Shishi Li, Yier Zhou, Weihai Xu, Jing Shu,Chongyi Shu

Signed for and on behalf of the Author(s):	Print Name:	Date:
Jing Shu	Jing Shu	2021-10-06
